# Clinically Relevant Immune Responses against Cytomegalovirus: Implications for Precision Medicine

**DOI:** 10.3390/ijms20081986

**Published:** 2019-04-23

**Authors:** Joana R. Lérias, Georgia Paraschoudi, Inês Silva, João Martins, Eric de Sousa, Carolina Condeço, Nuno Figueiredo, Carlos Carvalho, Ernest Dodoo, Elke Jäger, Martin Rao, Markus Maeurer

**Affiliations:** 1 ImmunoSurgery Unit, Champalimaud Centre for the Unknown, Av. Brasília, 1400-038 Lisbon, Portugal; joana.lerias@research.fchampalimaud.org (J.R.L.); georgia.paraschoudi@research.fchampalimaud.org (G.P.); ines.silva@research.fchampalimaud.org (I.S.); joao.martins@research.fchampalimaud.org (J.M.); eric.desousa@research.fchampalimaud.org (E.d.S.); carolina.condeco@research.fchampalimaud.org (C.C.); martin.rao@research.fchampalimaud.org (M.R.); 2 Digestive Unit, Champalimaud Centre for the Unknown, Av. Brasília, 1400-038 Lisbon, Portugal; nuno.figueiredo@fundacaochampalimaud.pt (N.F.); carlos.carvalho@fundacaochampalimaud.pt (C.C.); 3 Department of Oncology and Haematology, Krankenhaus Nordwest, Steinbacher Hohl 2-26, 60488 Frankfurt am Main, Germany; Ernest.Dodoo@protonmail.com (E.D.); EJ200161@aol.com (E.J.)

**Keywords:** cytomegalovirus, immune responses, interferon gamma, antibodies, cancer, immunotherapy, T cells

## Abstract

Immune responses to human cytomegalovirus (CMV) can be used to assess immune fitness in an individual. Further to its clinical significance in posttransplantation settings, emerging clinical and translational studies provide examples of immune correlates of protection pertaining to anti-CMV immune responses in the context of cancer or infectious diseases, e.g., tuberculosis. In this viewpoint, we provide a brief overview about CMV-directed immune reactivity and immune fitness in a clinical context and incorporate some of our own findings obtained from peripheral blood or tumour-infiltrating lymphocytes (TIL) from patients with advanced cancer. Observations in patients with solid cancers whose lesions contain both CMV and tumour antigen-specific T-cell subsets are highlighted, due to a possible CMV-associated “bystander” effect in amplifying local inflammation and subsequent tumour rejection. The role of tumour-associated antibodies recognising diverse CMV-derived epitopes is also discussed in light of anti-cancer immune responses. We discuss here the use of anti-CMV immune responses as a theranostic tool—combining immunodiagnostics with a personalised therapeutic potential—to improve treatment outcomes in oncological indications.

## 1. Introduction

Intact immune responses to cytomegalovirus (CMV) in humans are generally acknowledged as a marker of immunological fitness [1]. The immune system in patients undergoing solid organ or stem cell transplantation is strongly suppressed to avoid graft rejection, wherein a CMV infection and the ensuing disease pose a major clinical hurdle [2,3]. Congenital CMV infections contribute to central nervous system (CNS) pathology in approximately 10% of infants infected with CMV [4]. Another modality where CMV-related complications can be detrimental is a coinfection with the human immunodeficiency virus (HIV) [5].

CMV belongs to the *Herpesviridae* family, which also encompasses other viruses of clinical significance, i.e., Epstein–Barr virus (EBV; infectious mononucleosis, nasopharyngeal carcinoma, and B-cell lymphoma), varicella zoster virus (VZV; chicken pox), herpes simplex virus (HSV; cold sores and genital herpes), and Kaposi’s sarcoma-associated herpesvirus (KSHV, also known as HHV-8; Kaposi’s sarcoma and primary effusion lymphoma) [6]. Herpesviruses, including CMV and EBV, are known to establish latency in humans, the effect of which leads to unique and clinically relevant immunomodulation which—based on clinical and preclinical studies—stretches across the spectrum of immune-associated diseases to protective cellular immune responses in healthy individuals [6]. This review focusses on CMV and the associated clinically relevant immune responses.

CD8+ T cells are considered central to providing protective immune responses against CMV replication and disease [7] although emerging evidence suggests a role for natural killer (NK) cells with characteristics resembling immunological memory [8]. The T-cell receptor (TCR) repertoire for CMV epitopes, once established, has been shown to exist in an individual for several years although the differentiation status of the CMV-reactive T cells themselves may change over time [9]. CMV pp65 CD4+ T cells are enriched in the bone marrow of healthy individuals, forming a life-long immune reservoir [10]. Furthermore, mechanistic studies performed in mice established that this phenomenon is most likely due to competition between TCR subtypes recognising various immunodominant epitopes for clonal dominance in an individual’s anti-CMV memory T-cell pool [11]. Nevertheless, the *diversity* of the conventional anti-CMV TCR alpha beta (αβ) repertoire in a healthy individual is potentially more important compared to the frequency (thus, actual numbers) of virus-specific T cells or increased serum IgG titres to contain CMV [12] in order to keep infected cells under immune surveillance, reflected by CMV-specific TCR-β signatures in CMV-positive healthy individuals who contain the infection [13].

The phenomenon of inducing and maintaining a general state of systemic inflammation marked by upregulated levels of pro-inflammatory cytokines (i.e., IL-18, IL-6, IP-10, TNF-α, and IFN-γ) in serum during latency after a primary infection is also a characteristic of CMV, as shown not only in the context of renal transplant recipients [14] but also in CMV-positive, healthy humans [15,16]. CMV-driven inflammation—if not overt—maybe beneficial in potentiating general immune surveillance and control in the host, such as in cancer [17] and drug-susceptible pulmonary tuberculosis [18]. 

## 2. CMV–Host Interactions in Cancer

Although anti-CMV immune responses appear to favour the immune control of cancer, a possible link with a CMV infection may underlie tumour progression and immunopathogenesis. For example, an association between a CMV infection and glioblastoma multiforme (GBM) has been substantiated with the isolation of viral nucleic acids and proteins from GBM lesions [19,20,21] as well as an improvement of patients with GBM following antiviral (valganciclovir) therapy [22,23,24]. Furthermore, CMV pp65-specific T cells are able to recognise and kill GBM cells, potentially with the ability to prolong the survival of patients with GBM [25,26], further strengthening the case for CMV involvement in GBM development in humans. This is further evidenced by several clinical trials which are underway, either based on or incorporating the use of anti-CMV immune responses as a therapeutic tool to improve clinical outcomes for patients with GBM (ClinicalTrials.gov identifiers: NCT02661282; NCT03615404; NCT00639639; NCT02864368; and NCT01109095). However, formal testing in a suitable clinically relevant model is necessary to investigate whether a CMV infection directly causes malignant transformation. Some indirect evidence supports the notion that a CMV infection may facilitate cancer-cell invasiveness, associated with CMV-driven cell-surface adhesion molecule expression dynamics [27] and increased tyrosine kinase activity [28]. A CMV infection and oncogenesis has also been associated with colorectal [29] and breast cancers [30] based on increased antibody levels in serum and tumour tissue testing positive for virus-derived materials. 

A link between immune checkpoint inhibitor (ICI)-induced colitis in forty-one patients with melanoma and CMV infection has also been reported [31]. Five of the forty-one patients (4 received ipilimumab alone; 1 received ipilimumab + nivolumab) exhibited treatment-refractory colitis and showed titres of anti-CMV IgG and/or IgM in serum, while eight patients with non-refractory colitis only had IgG titres, suggesting that a recent CMV infection or reactivation from latency may be a comorbidity in patients who succumb to treatment-resistant colitis following ICI therapy. Furthermore, the patient with treatment-refractory colitis who received a combination of ipilimumab and nivolumab did not exhibit CMV-directed IgM in serum and no evidence of CMV proteins was observed based on an immunohistological analysis of the inflamed colon biopsy despite testing positive for viral DNA in stool, blood (serum), and colon tissue. 

Another, more comprehensive immunological study, involving a single patient with metastatic melanoma who received ipilimumab + nivolumab, showed that CMV colitis, which was successfully controlled using antivirals (ganciclovir and valganciclovir), combined with corticosteroids and anti-TNF-α therapy resulted in an increased anti-CMV serum IgG as well as an infiltration of NK and CD8+ T cells into the colon [32]. The authors also observed an increase in T-cell chemoattractants, i.e., CXCL9 and CXCL10 following CMV clearance, suggesting an improved immune surveillance and enhanced CMV control following the development of autoimmune responses. 

The clinical use of anti-CMV intravenous immunoglobulin gamma (CMV-IVIG) preparations has been evaluated in patients receiving haematopoietic stem cell transplantation (HSCT) as well as solid organ transplantation, with good safety profiles as well as recommendations for an early initiation of treatment in some cases [33,34,35]. Although CMV-IVIG is aimed at neutralising viral titres in systemic circulation, one may argue that some of the IgG would traffic to organs and mediate antiviral activity locally due to its ability to ameliorate symptoms and to improve patients’ performance during tissue-invasive CMV disease in the background of resistance to ganciclovir/valganciclovir [36]. CMV- and EBV-specific antibodies produced by tumour-infiltrating B cells (TIBs) have been characterised using a peptide-target microarray platform to evaluate the recognition pattern of viral epitopes within the tumour as compared to a CMV target recognition of antibodies in serum [37]. Interestingly, TIB-derived IgG molecules displayed the widest diversity of CMV/EBV epitope recognition, while the number of targets recognised by serum- and TIB-derived IgG were comparable. Thus, while the amount of IgG secreted by TIB locally within the tumour remains unknown, many tumour-associated CMV pp65 epitopes can evoke an IgG response. In this regard, whether intratumoural IgG production suggests a possible augmentation of epitope-specific ADCC in the tumour or, rather, virus neutralisation requires formal testing, these data reflect the existence of circulating memory T and B cells which can respond to CMV pp65-derived epitopes. 

Unconventional TCR γδ T cells initially induced by a CMV reactivation in patients receiving HSCT were able to recognise and eliminate leukaemia cells after transplantation [38]. Also, an expansion of PBMCs with IL-2, IL-15, OKT3, and IFN-γ in addition to CMV pp65 stimulation has been shown to generate a unique population of CD3+ CD8+ CD56+ T cells with cytotoxic properties capable of killing leukaemia cells in vitro [39]. The TCRs which can be isolated from blood may be tested for their composition and structure, i.e., Vαβ/γδ sequences and their complementarity-determining region 3 (CDR3) patterns using deep TCR sequencing [40], which would also allow the use of these molecularly defined TCRs as “blueprints” for transgenic expression in appropriate recipient cells. These immune cells, expressing the nominal TCR as a transgene, can be used to identify the nominal target antigen(s). Similarly, the TCRs of TIL as well as BCRs and IgG derived from TIBs which can recognise CMV epitopes are viable candidates for a more detailed, structural B- and T-cell composition analysis. A detailed TCR analysis, using deep TCR sequencing in T cells reacting either to pp65495–503 (NLV) of CMV and the M158–66 (GIL) of the influenza A virus revealed a highly diverse TCR repertoire, restricted by preferential patterns within the CDR3, where TCR specificity resides, suggesting a broad TCR repertoire against individual dominant HLA-A2 restricted epitopes [41].

### T-Cell Reactivity Against CMV Targets in T Cells from Patients with Cancer—Experience at the ImmunoSurgery Unit, Champalimaud Centre for the Unknown (CCU)

**Figure 1:**
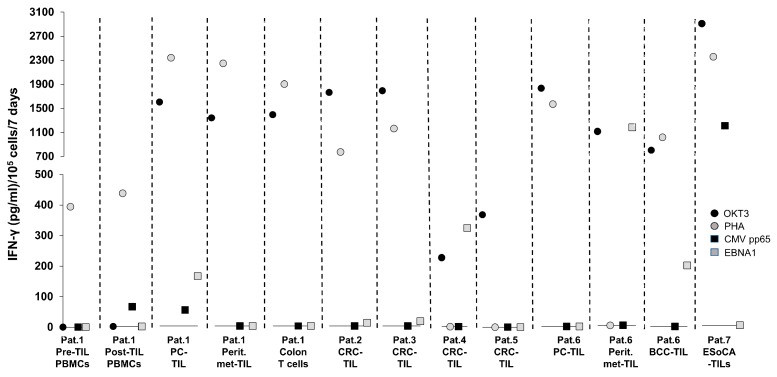
The anti-CMV/EBV cellular immune responses of T cells derived from patients with cancer: T-cell preparations sourced from whole PBMCs or tumour-infiltrating lymphocytes (TIL) of patients with various diagnoses of gastrointestinal malignancies were tested in a 7-day in vitro stimulation assay with CMV pp65 or EBV-EBNA1. Cell culture supernatants were harvested for IFN-γ measurement by sandwich ELISA. As positive controls, the antihuman CD3 antibody (clone OKT3) as well as the mitogen phytohaemagglutinin (PHA) were used separately to ascertain maximal T-cell responses. The antigen-driven cellular immune response is expressed as IFN-γ production per 1 × 10^5^ cells over 7 days. Legend: Pat.1–7 = patients 1–7; TIL = tumour-infiltrating lymphocytes; PBMCs = peripheral blood mononuclear cells; PC = pancreatic cancer; CRC = colorectal cancer; perit.met = peritoneal metastasis; BCC = basal cell cancer (basalioma); EsoCA = oesophageal cancer.

We have observed that some patients with gastrointestinal malignancies can mount a strong anti-CMV/EBV T-cell response following viral antigen stimulation. As shown in Figure 1, we tested the immune reactivity of T cells from 7 patients presenting with gastrointestinal epithelial cancers (2 metastatic pancreatic cancer, 4 colorectal cancer, and 1 oesophageal cancer). Descriptions of the samples tested in the assay are shown in Table 1.

One of the patients diagnosed with metastatic pancreatic cancer also presented with basal-cell cancer (basalioma), from which TIL were isolated and cultured for *in vitro* studies. T cells were expanded from TIL preparations used for clinical therapy (Patient 1) for immunological analysis for research purposes (Patients 2–7), from PBMCs for immune-monitoring post-TIL therapy (Patient 1), and from noncancerous tissue-derived material (inflamed colon tissue from Patient 1). A 7-day in vitro stimulation assay in the presence of the CMV pp65 and EBV-EBNA1 peptides along with the appropriate positive controls (antihuman CD3 antibody OKT3 or phytohaemagglutinin, PHA), which is routinely performed to evaluate the immune status/‘immune-fitness’ of the patients, showed that the T-cell samples used in the assay were immunologically responsive based on their activation by OKT3 and/or PHA. An antiviral IFN-γ production of TIL isolated from the primary tumours of several patients, i.e., pancreatic cancer TIL from Patient 1 (anti-CMV and anti-EBV reactivity), CRC-TIL from Patient 4 (anti-EBV reactivity only), BCC-TIL from Patient 6 (anti-EBV reactivity only), and oesophageal cancer TIL from Patient 7 (anti-CMV reactivity only), was observed. While patients 1 and 6 had metastatic pancreatic cancer that spread to the peritoneum, only Patient 6 showed a strong anti-EBV response in metastasis-derived TIL. Patient 1, who received a TIL infusion as immunotherapy, did not have anti-CMV/EBV cellular immune responses in blood prior to TIL therapy but developed measurable anti-CMV reactivity afterwards, suggesting increased antiviral immune responses potentiated by T-cell therapy. Not mutually exclusively, CMV may have also been (re)activated before TIL therapy due to the lymphodepletion prior to T-cell infusion—with a subsequent expansion of CMV-specific T cells. These findings may be clinically relevant because (i) the host’s cellular immune system can recognise CMV/EBV targets in different organs/tissues, i.e., in the gut (pancreas, peritoneum, and colorectum); (ii) these immune responses may be related to the patients’ clinical performance during chemotherapy, surgery, and/or immunotherapy; and (iii) some of these immune responses may be clinically and biologically relevant, since EBV/CMV-reactive T cells among TIL may change the *milieu interne* of the tumour microenvironment (TME) towards a more productive, anticancer Th1-type response. Cytokines produced by CMV-specific T cells (e.g., IFN-γ) may upregulate the antigen processing and presentation machinery in tumour cells and may increase the capacity of tumour cells to present tumour-associated targets to cancer-directed T cells. 

CMV involvement in gastrointestinal pathologies have been reported: CMV-associated oesophagitis in an immunocompetent individual who underwent short-term corticosteroid therapy [42] and CMV-related pancreatitis and hepatitis have been previously reported in an immunocompetent individual [43]. The actual role of the strong anti-CMV immune responses in oesophageal and pancreatic cancer TIL seen in Patient 7 requires further biological dissection. Similarly, a link between EBV and CRC was recently reviewed by Bedri and colleagues [44], wherein several lines of evidence based on EBV nucleic acids and proteins, but not T-cell responses in patient samples, were summarised. CMV-directed T cells have been shown to infiltrate primary melanoma lesions in mice and maintain immune reactivity despite PD-1 expression [45]. A similar phenomenon may also exist in humans, based on the EBV reactivity of BCC TIL shown in Figure 1.

Mechanistically, CMV has been shown to induce changes in endothelial cells by promoting their motility and propensity for neovascularisation, achievable by a direct binding of the virus to the endothelial growth factor receptor (EGFR) on the cell surface [46]. Alternatively, CMV-infected host cells are induced to produce vascular endothelial growth factor (VEGF) which is necessary for the formation of new blood vessels [47]. VEGF-mediated aberrant neovascularisation is a major contributing factor to hypoxia (caused by the activation of hypoxia-inducing factor 1 alpha, HIF-1α) and a reduced susceptibility of transformed cells to chemotherapy in cancer [48] as well as in relation to granuloma-restricted *Mycobacterium tuberculosis*-infected cells in pulmonary tuberculosis [49]. CMV-induced IL-6 production has also been linked to neovascularisation, with a role for the cytokine-driven upregulation of survivin, an apoptosis-inhibiting protein and a known tumour-associated antigen (TAA) in pancreatic cancer and glioblastoma which is also implicated in antitumour-immune responses [50,51,52]. While CMV-mediated immune responses might reflect immune fitness, the hypothesis that CMV infection may facilitate a malignant transformation of host cells requires formal testing using suitable, clinically relevant models.

## 3. Harnessing Anti-CMV Immunomodulation for Precision Oncology

CMV- and EBV-specific T-cell responses in patients with pancreatic or brain cancer are able to predict a better survival pattern [17], akin to what has been described in patients with drug-susceptible pulmonary tuberculosis who undergo standard antibiotic therapy [18]. The consensus is that regardless of CMV infection status, the presence of anti-CMV T cells in peripheral blood is indicative of better survival among patients with cancer. Inflationary CMV-specific CD8+ T cells can proliferate extensively in the host following antigenic stimulation and can ‘over-crowd’ the total pool of memory T cells in the host. Some of these cells may, in fact, provide protection to other diseases, i.e., intracellular infections, or, on the contrary, drive inflammation-induced tissue destruction [53]. Active CMV infection has also been shown to dominate the antigen processing machinery in host cells, with most of the intracellular HLA class I molecules loaded with CMV pp65-derived epitopes [54]. Simultaneously, CMV-specific CD8+ T cells may play a role in providing antitumour responses in situ [55]. TIL from patients with lung or colorectal cancer have been shown to contain HLA-A2- and HLA-24-restricted CMV pp65- and EBV-specific CD8+ T cells which are devoid of CD39 expression, an extracellular ATP-catabolising enzyme that is associated with an anti-inflammatory skewing of immune responses [56,57]. Interestingly, the maintenance of tissue-resident inflationary CMV-specific CD8+ T cells requires IL-15 trans-presentation [58], which is also involved in promoting antitumour surveillance mediated by memory CD8+ T cells [59,60,61]. These antiviral TIL may participate in the antitumour response as “bystander” immune cells although not directly reacting to tumour-derived antigens. 

An examination of CMV-specific CD8+ T-cell infiltration into melanoma lesions in a mouse model showed that the expression of PD-1 did not block them from performing effector functions i.e., producing IFN-γ [45]. PD-1+ CD8+ T cells responding to viral antigens were found to exist in the tumour lesions even in the background of a chronic infection with MCMV (virus-infected mice which were further challenged with B16F0 cutaneous melanoma). In agreement with this observation, effector memory CD8+ TIL from human GBM lesions, albeit expressing CTLA-4, were able to maintain their degranulation potential (CD107+ induction) following exposure to CMV pp65 or IE-1, another immunodominant CMV antigen [62]. Also, these T cells were found to infiltrate GBM tumours which had a high expression of the IE-1 protein, suggesting antigen-driven chemoattraction *in situ*. Another translational study, using mononuclear cells isolated from pleural effusion (PE) in patients with lung cancer, showed that a PD-1 expression on PE-derived CMV-pp65-specific CD8+ and CD4+ T cells retained their capacity to express CD137 (4-1BB) in response to viral antigen stimulation [63]. However, PD-1+ T cells, that also co-express TIM-3, displayed a diminished antigen responsiveness, indicating the need for a co-expression of alternate immune checkpoints for assessing immunological fitness. Therefore, anti-CMV T cells—albeit expressing PD-1 and being present in tumours—are not functionally compromised and may produce clinically relevant immune responses in situ. Indeed, Scheper and colleagues recently showed that CMV- and EBV-specific T cells can be found in human ovarian and colorectal carcinoma lesions [64]. Nevertheless, virus-specific TCRs isolated from 2 of the patients with microsatellite-stable CRC—upon transfection into healthy donor-derived (allogeneic) peripheral blood T cells—could not recognise and kill the patients’ tumour cell-derived organoids [64].

**Figure 2:**
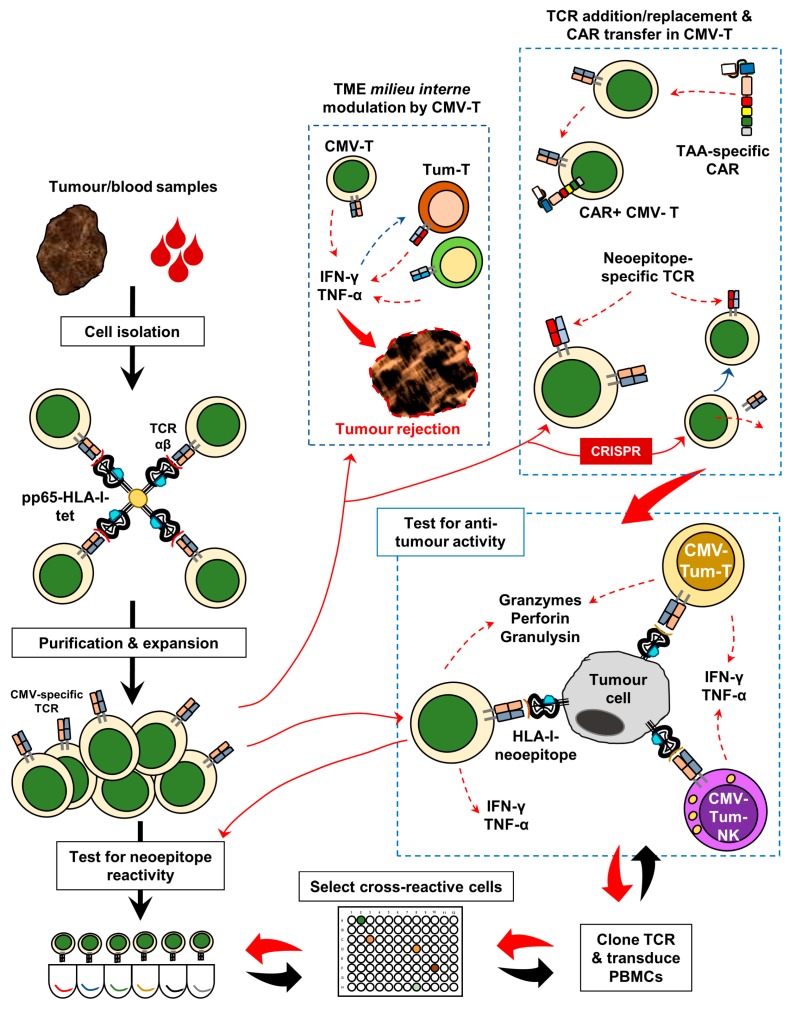
The CMV-based precision immunotherapy development: A potential strategy to isolate CMV- and tumour-reactive TCRs from tumour tissue and/or blood samples for developing targeted T cell-based therapies using CMV-pp65 as a model antigen as described in the main text. Further, to infiltrating T cells, “bystander” CMV-specific T cells in the TME via the local production of IFN-γ and TNF-α, may trans-activate endogenous TIL with neoepitope TCRs to jointly enhance the antitumour response and potentiate tumour rejection. Legend: CMV-T = CMV-specific T cells; TCR = T-cell receptor; pp65-HLA-I-tet = HLA class I tetramer; CMV-Tum-T = CMV-tumour neoepitope-specific TCR-transferred T cell; CMV-Tum-NK = CMV-tumour neoepitope-specific TCR-transferred NK cell; CAR = chimeric antigen receptor; CRISPR = clustered regularly interspaced short palindromic repeats; IFN-γ = interferon gamma; TNF-α = tumour necrosis factor alpha.

Based on clinical observations and a translational evaluation of patient material as well as some mechanistic studies in animal models, it remains to be established whether CMV-derived epitopes are presented by HLA molecules of tumour cells. If so, the next step would be to determine whether these epitopes correspond to antitumour activity. Not mutually exclusively, CMV-specific TCRs may—due to molecular mimicry—recognise cancer-associated neoepitopes in some individuals. This could be formally tested to measure the potential cross-reactivity with tumour-related antigenic targets, i.e., peptide sequences predicted based on whole-exome and RNA sequencing. Thus, a possible cross-reactivity may also suggest that specific CMV-directed TCRs (and possibly BCRs) may also participate in inducing antitumour responses. Figure 2 is a schematic representation of a possible strategy which can be pursued for using CMV-tumour neoantigen-cross-reactive TCRs for a personalised immunotherapy of cancer. These TCRs can be obtained from blood or tissue, i.e., tumour biopsies or surgical resections. CMV-pp65-specific CD8+ T cells, for instance, can be isolated from PBMC or TIL preparations using HLA class I tetramers, expanded in vitro in the presence of specific cytokines (e.g., IL-2, IL-15, IL-21, and IL-7), and then tested for reactivity with neoantigens and/or tumor-associated non-mutated targets. The TCRs of interest can be transferred to nonspecific T cells or natural killer (NK) cells using lentiviral expression systems. Alternatively, CRISPR technology can also be used for replacing the (CMV-reactive) TCR with a neoantigen-reactive counterpart, mainly due to the (i) intrinsic immune effector characteristics of the cell itself or (ii) to avoid competition of the mixed-dimer formation of anti-CMV/anticancer antigen TCR chains [65]. Chimeric antigen receptor (CAR) constructs can also be transferred to CMV-specific T cells, subsequently endowing them with the capacity for both HLA-dependent target recognition (TCR-based) as well as a CAR-mediated binding of TAAs on target cells to elicit antitumour effects in the TME (tumor microenvironment). Furthermore, CMV-specific T cells infiltrating into tumour lesions produce a pro-inflammatory milieu that upregulates HLA molecules, facilitates Th1/Tc1 responses, and may help expand “private” tumour antigen-specific T cells. This in trans stimulation of T cells—unrelated to CMV targets—may receive cytokine signals from CMV-specific “bystander” T cells, which help amplify the local antitumour immune response [57]. It may not be critical whether the transformed, non-transformed, as well as stromal cells in the TME are infected with CMV. 

Independent of whether bona fide cross-reactivity exists between CMV antigenic targets and neoepitopes, CMV-reactive T cells homing to CMV epitope-expressing tumour tissue or non-transformed stromal tissue could represent a long-lived immune-recipient cell producing antitumour pro-inflammatory cytokines, either for transgenic TCRs directed against TAAs or CARs [66]. Thus, CMV-specific T cells present a highly resourceful and versatile source of antitumour-directed cellular immune responses which can be used in immuno-oncology clinical protocols for patients who are able to mount a strong CMV-directed immune response and provide, therefore, an autologous source of long-term memory and immune effector T cells. 

## 4. Material and Methods (Original Data from the CCU)

### T-Cell Exposure to CMV pp65 and EBNA-1 Peptides

TIL-formed tumour lesions were isolated and expanded in the laboratory as previously described [51,67]. PBMCs from patients were isolated after separating whole venous blood over a Ficoll–Hypaque gradient (GE Healthcare, Uppsala, Sweden). The cells were then washed twice with sterile PBS prior to use in cell culture experiments. T cells (1.0 × 10^5^) from TILs/PBMCs were cocultured with 1 μg of CMV peptides in 96-well tissue culture plates containing 200 μL of a T-cell medium (GellGenix^®^ GMP-DC medium + recombinant human cytokines; IL-2, 1000 IU/mL; IL-15, 180 IU/mL; IL-21, 10 IU/mL + 10% human AB serum). Wells with the T-cell medium alone were used as negative controls, while 2 wells with 30 ng/mL of antihuman CD3 antibody (clone OKT3, Biolegend, San Diego, CA) or 5 μg/mL of phytohaemagglutinin (Sigma-Aldrich, St. Louis, MO, USA) for maximal stimulation were used as positive controls. After incubating the cells with peptides for 7 days at 37 °C with 5% CO_2_, culture supernatants were harvested for IFN-γ sandwich ELISA. The IFN-γ data is reported as follows: IFN-γ (pg)/7 days/1.0 × 10^5^ T cells, after subtracting peptide-specific response values from the negative control (T-cell medium only) values. 

## 5. Conclusions

The use of CMV-specific T cells and/or IgG to treat patients with a viral reactivation disease in the post-transplant setting, or in the context of CMV pp65-targeted dendritic cell vaccination provides solid ground for the safety of CMV-based immunotherapy. As discussed in this review, future clinical studies should aim to decipher the role of CMV in various cancer settings—understanding whether viral infection is directly associated with oncogenesis. Targeting CMV-directed immune responses to malignant lesions may also provide acytokine milieufacilitating productive anti-tumour immune responses even without the need for CMV target expression on transformed cells- Such a ‘Th1-wired’ immune responset could help to expand tumour antigen-specific T cells. 

## Figures and Tables

**Table 1:** Descriptions of the clinical samples used in the cytomegalovirus (CMV)-Epstein–Barr virus (EBV)-immunoreactivity assay.

Patient	Description	Samples Tested for CMV/EBV Reactivity
1	Metastatic pancreatic cancer (PC), peritoneal spread. Patient received TIL therapy. A colon biopsy was taken due to a *clostridium difficile* infection one month post-TIL therapy. PBMCs were sampled before and after TIL infusion.	PBMCs before TIL infusion PBMCs after TIL infusion Pancreatic cancer TIL TIL from peritoneal metastasis Colon-derived T cells
2	Colorectal cancer (CRC)	TIL from CRC tissue
3	CRC	TIL from CRC tissue
4	CRC	TIL from CRC tissue
5	CRC	TIL from CRC tissue
6	Metastatic pancreatic cancer which spread to the peritoneum. Patient also had basal-cell carcinoma (BCC/basalioma).	Pancreatic cancer TIL TIL from peritoneal metastasis TIL from BCC tissue
7	Oesophageal cancer (EsoCA)	TIL from EsoCA tissue

## Abbreviations

CCU	Champalimaud Centre for the Unknown
CMV	Cytomegalovirus
EBV	Epstein–Barr virus
MCMV	Murine cytomegalovirus
TIL	Tumour-infiltrating lymphocytes
TIB	Tumour-infiltrating B cells
PBMCs	Peripheral blood mononuclear cells
TCR	T-cell receptor
BCR	B-cell receptor
HLA	Human leukocyte antigen
MHC	Major histocompatibility complex
TME	Tumour microenvironment
PD-1	Programmed cell death 1
TIM-3	T-cell immunoglobulin mucin-domain-containing 3
IgG	Immunoglobulin gamma
HSCT	Haematopoietic stem cell transplantation
ADCC	Antibody-dependent cellular cytotoxicity
WBA	Whole-blood assay
ELISA	Enzyme-linked immunosorbent assay
IFN-γ	Interferon gamma
pg	picogrammes
IU	International units
GBM	Glioblastoma multiforme
PC	Pancreatic cancer
CRC	Colorectal cancer
EsoCA	Oesophageal cancer
BCC	Basal cell carcinoma
ICI	Immune checkpoint inhibitor
CMV-IVIG	Cytomegalovirus-intravenous immunoglibulin
PE	Pleural effusion
EBNA-1	Epstein–Barr nuclear antigen 1
PHA	Phytohaemagglutinin
CAR	Chimeric antigen receptor
Th1	T helper 1
